# Prevalence and predictors of healthcare use for psychiatric disorders at 9 years after a first episode of psychosis: a Swedish national cohort study

**DOI:** 10.1136/bmjment-2024-301248

**Published:** 2025-03-26

**Authors:** Donna van Deursen, Ellenor Mittendorfer-Rutz, Heidi Taipale, Emma Pettersson, Philip McGuire, Paolo Fusar-Poli, Dan W Joyce, Nikolai Albert, Annette Erlangsen, Meredete Nordentoft, Carsten Hjorthøj, Simon Cervenka, Alexis E Cullen

**Affiliations:** 1 Division of Insurance Medicine, Department of Clinical Neuroscience, Karolinska Institutet, Stockholm, Sweden; 2 Niuvanniemi Hospital, Kuopio, Finland; 3 School of Pharmacy, University of Eastern Finland, Kuopio, Finland; 4 Department of Psychiatry, Division of Medical Sciences, University of Oxford, Oxford, UK; 5 Department of Brain and Behavioral Sciences, University of Pavia, Pavia, Italy; 6 Early Psychosis: Interventions and Clinical-detection (EPIC) Lab, Department of Psychosis Studies, Institute of Psychiatry, Psychology & Neuroscience, King’s College London, London, UK; 7 Outreach and Support in South-London (OASIS) service, South London and Maudsley (SLaM) NHS Foundation Trust, London, UK; 8 Department of Psychiatry and Psychotherapy, Ludwig-Maximilian-University Munich, Munich, Germany; 9 Institute of Population Health, University of Liverpool, Liverpool, UK; 10 Copenhagen Research Centre for Mental Health—CORE, Copenhagen, Denmark; 11 Department of Clinical Medicine, Faculty of Health and Medical Sciences, University of Copenhagen, Copenhagen, Denmark; 12 Danish Research Institute for Suicide Prevention, Hellerup, Denmark; 13 Department of Public Health, Section of Epidemiology, University of Copenhagen, Kobenhavn, Denmark; 14 Centre for Psychiatry Research, Department of Clinical Neuroscience, Karolinska Institutet, Stockholm, Sweden; 15 Department of Medical Sciences, Psychosis Research and Preventive Psychiatry, Uppsala University, Uppsala, Sweden; 16 Department of Psychosis Studies, Institute of Psychiatry, Psychology & Neuroscience, King’s College London, London, UK

**Keywords:** Schizophrenia & psychotic disorders, Adult psychiatry

## Abstract

**Background:**

Psychotic disorders are known to exhibit heterogeneity with regards to illness course and prognosis, yet few studies have examined long-term healthcare use.

**Objective:**

To determine the prevalence and predictors of healthcare use for psychiatric disorders at 9 years after the first episode of psychosis (FEP).

**Methods:**

National registers were used to identify all Swedish residents aged 18–35 years with FEP between 2006 and 2013. The 12-month period-prevalence of secondary healthcare use was determined at each year of the 9-year follow-up, categorised according to main diagnosis (psychotic disorder vs other psychiatric disorder vs none vs censored). Multinomial logistic regression models were used to examine associations between baseline characteristics and healthcare use at 9 years and derive predicted probabilities and 95% CIs for the four outcome groups, for each predictor variable.

**Findings:**

Among 7733 individuals with FEP, 31.7% were treated in secondary healthcare for psychotic disorders at the 9-year follow-up, 24.1% were treated for other psychiatric disorders, 35.7% did not use healthcare services for psychiatric disorders and 8.5% were censored due to death/emigration. Having an initial diagnosis of schizophrenia was associated with the highest probability of secondary healthcare use for psychotic disorder at 9 years (0.50, 95% CI (0.46 to 0.54)] followed by inpatient treatment at first diagnosis (0.37, 95% CI (0.35 to 0.38)).

**Conclusion:**

Although 56% of individuals with FEP were treated for psychiatric disorders in secondary healthcare 9 years later, a substantial proportion were treated for non-psychotic disorders.

**Clinical implications:**

Individuals with an initial diagnosis of schizophrenia, who received their first diagnosis in inpatient settings, may need more intensive treatment to facilitate remission and recovery.

**WHAT IS ALREADY KNOWN ON THIS TOPIC**

- The extent to which individuals experiencing a first episode of psychosis (FEP) continue to receive treatment in secondary healthcare services for psychiatric disorders in the mid-term to long-term is unknown.

**WHAT THIS STUDY ADDS**

- In a nationwide cohort of all individuals diagnosed with FEP from 2006 to 2013, one-third (31.7%) used secondary healthcare for psychotic disorders 9 years later and a further 24.1% were treated for other psychiatric disorders.
- Individuals who had an initial diagnosis of schizophrenia, those who were first diagnosed in inpatient care, and individuals born outside of Sweden had the highest probabilities of receiving treatment for psychotic disorders at 9 years after the first diagnosis.

**HOW THIS STUDY MIGHT AFFECT RESEARCH, PRACTICE OR POLICY**

- This real-world evidence study indicates that the healthcare burden of psychotic disorders is high, with around 56% of individuals with FEP being treated for psychiatric disorders at 9 years after onset.
- Specific diagnostic and population subgroups appear to be at greater risk of long-term healthcare utilisation and may therefore require additional support to facilitate remission and recovery.

## Background

 Psychotic disorders constitute a heterogeneous group of psychiatric conditions, which differ in terms of their aetiology, symptom presentation and illness course. These disorders can be broadly categorised as ‘primary’ or non-affective (eg, schizophrenia, schizophreniform disorder, acute and transient psychotic disorder, schizoaffective disorder, delusional disorder, other psychotic disorders) versus affective (bipolar and depressive disorders with psychotic features), with the former characterised by higher levels of positive symptoms, a longer duration of untreated psychosis, worse cognitive performance and poorer social and occupational outcomes.^1^,^3^ Many individuals with primary psychotic disorders experience a prodromal period,^4^ characterised by subthreshold psychotic symptoms, before transitioning to full-threshold disorder; however, others have a rapid onset of acute illness. Although it is possible to make a full recovery after the first episode of psychosis (FEP), some people will experience relapses, while for others (particularly those with schizophrenia), illness is severe and unremitting.^5 6^ Given the huge impact that these disorders can have at the personal, societal and economic level, there have been increased efforts to improve long-term outcomes by intervening during the early course of illness^7^ and identifying robust prognostic markers.^8^

Studies examining long-term outcomes among individuals with FEP have largely focused on remission (symptomatic and/or functional improvement) and recovery (encompassing improvement in symptoms, social and occupational functioning and subjective experiences of psychological well-being). Recent meta-analyses have reported overall remission and recovery rates of 54% and 32%, respectively, among individuals with FEP when both non-affective and affective subtypes are included,^9^ but lower recovery rates (21%) when restricted to the former.^6^ Far less is known about the need for long-term healthcare utilisation in FEP populations, irrespective of subtype. This knowledge is of high importance to clinical teams and healthcare planners. Indeed, direct healthcare costs have been shown to account for a substantial proportion of the societal costs associated with schizophrenia.^10^

Studies examining long-term healthcare utilisation among individuals with FEP (including non-affective and affective subgroups) have largely focused on inpatient care. Such studies show that after the first contact for FEP, between 50% and 70% of individuals experience at least one hospitalisation within 5–10 years.^11^,^13^ Use of outpatient services has been less commonly investigated; however, 5-year follow-up data from a randomised trial in Denmark found that around half of patients (irrespective of whether they had been treated in specialist early intervention services) had attended outpatient clinics in the last year.^14^ Factors such as gender, ethnicity and diagnostic group have not been consistently associated with long-term healthcare utilisation, which may in part be explained by differences in study design. As such, we have limited knowledge regarding the extent to which individuals with FEP continue to use healthcare services for psychiatric disorders in the medium-to long-term and which factors increase the probability of this outcome.

To address these knowledge gaps, the present study used data from a well-characterised cohort of all individuals in Sweden aged 18–35 years, who were treated for the first time for a primary (non-affective) psychotic disorder from 2006 to 2013.^15 16^ This age range, which includes the peak age of onset for these disorders,^17^ was selected to minimise potential heterogeneity in illness course related to age-of-onset^18^,^20^; affective psychoses were not included as these disorders are not typically treated in psychosis services in Sweden. National registers were used to determine healthcare use for psychiatric disorders during the first 9 years of illness and to measure a range of baseline factors, including sociodemographic characteristics, indices of functional impairments impacting work ability and clinical factors. The aims of the present study were to determine yearly proportions of individuals treated for psychotic disorders and other psychiatric disorders during the study period and to identify baseline factors associated with healthcare use for these diagnostic categories at 9 years after the first diagnosis.

## Methods

### Study design and data sources

This population-based cohort study used data from the Swedish national registers listed in online supplemental table 1. Registers were linked by the (pseudonymised) unique personal identification number assigned to all Swedish residents at birth or immigration. According to current Swedish regulations, the use of national data for research purposes does not require informed consent from individuals held in these registers.^21^ This article follows the REporting of studies Conducted using Observational Routinely-collected health Data Statement,^22^ and a study protocol was registered prospectively (https://osf.io/edjcs/).

### Study population

The study population was defined using the National Patient Register (NPR) according to the International Classification of Diseases—V.10 (ICD-10)^23^ code assigned as the main diagnosis at discharge or physician contact for inpatient and outpatient treatments, respectively. Consistent with our previous publications,^15 16^ we first identified all individuals aged 18–35 years residing in Sweden who received their first diagnosis of a primary (non-affective) psychotic disorder (ICD-10 codes: F20–F29) in inpatient/outpatient settings between the 1 January 2006 and 31 December 2013. First-episode cases were defined as having no inpatient/outpatient contacts for non-affective psychotic disorders in the previous 3 years (1080 days). All individuals were, therefore, required to have lived in Sweden for at least 3 full calendar years prior to inclusion. We additionally excluded individuals who had any recorded purchases of antipsychotic medications (Anatomic Therapeutic Chemical classification codes N05A, omitting lithium N05AN) in the Prescribed Drug Register. Because this register is available from July 2005, we used an exclusion period of 15–3 months prior to cohort entry for antipsychotic purchases (providing a minimum observation period of 6 months for all cohort members). Antipsychotic purchases during the 3 months prior to inclusion were permitted as we considered these to be part of the first treatment episode; however, for comparability, the first contact with inpatient/outpatient services was used as the inclusion date for all individuals. Cohort members were followed from the date of their FEP diagnosis for 9 years or until death/emigration.

### Exposure measures

Sociodemographic variables (age, sex, country of birth, family situation, type of residence area, level of education and unemployment), measures of work disability (sickness absence and work disability) and clinical factors (prior secondary healthcare use for any psychiatric disorder, prior psychotropic medication, diagnosis at cohort entry and treatment setting at FEP diagnosis) were determined using the Swedish registers detailed in online supplemental table 1.

### Outcome measures

Our primary outcome measure was secondary healthcare utilisation for psychiatric disorders, determined using the main ICD-10 diagnosis assigned to each inpatient/outpatient treatment in the NPR. These were categorised as healthcare contacts for psychotic disorders (F20–F29) versus other psychiatric disorders (all F codes excluding F20–F29) versus no healthcare contacts for any psychiatric disorder (no F codes recorded as a main diagnosis). The NPR includes all Swedish specialised care contacts, irrespective of setting (ie, all psychiatric and other medical services), with up to 30 diagnoses (1 main+29 side diagnoses) recorded for each contact. We used only the main diagnosis assigned to each contact to capture treatments for psychiatric disorders. Therefore, the ‘no secondary healthcare use for psychiatric disorders’ group potentially includes individuals who received specialised healthcare (psychiatric or other medical), but where the main diagnosis was not a psychiatric disorder as well as those who did not access secondary healthcare services (either because they were treated exclusively in primary care or because they did not receive healthcare in any service for psychotic disorders).

On the assumption that all individuals with an active (non-remitted) psychiatric disorder will likely receive some form of secondary care treatment during any 12-month period, we determined the 12-month period prevalence of secondary healthcare utilisation during each year of the 9-year follow-up. That is, for each 360-day period after the FEP diagnosis date, we examined all secondary healthcare contacts for psychiatric disorders occurring during that period. Because individuals could have more than one healthcare contact during any 12-month period (and so could potentially receive more than one psychiatric diagnosis), a hierarchical categorisation was applied at each year of follow-up, where psychotic disorders were prioritised over other psychiatric disorders, which was prioritised over no psychiatric disorder. In supplementary analyses, we also examined contacts for individual diagnoses including schizophrenia (ICD-10 code F20), acute/transient psychotic disorder (F23), other psychotic disorders (F21–F22, F24–29), bipolar (F30–F31), depressive (F32–F33), anxiety (F40–F41), stress-related (F43) and substance use disorders (F10–F19). In these supplementary analyses, categories were not mutually exclusive (ie, all diagnoses received during each 12-month period were included with no hierarchy applied).

### Statistical analyses

Statistical analyses were performed in R V.4.3.1. Patterns of healthcare utilisation for psychiatric diagnostic categories at each year after inclusion were visualised with a Sankey diagram created using the SankeyMATIC tool (https://sankeymatic.com/). In this descriptive analysis, which shows movement of individuals across the three broad diagnostic categories over time, individuals who were censored (due to death or emigration) were included as a separate trajectory group. In supplementary analyses, we examined healthcare contacts for individual diagnoses (where diagnostic categories were not mutually exclusive), visualised using partial density plots. These plots show, for each diagnostic category, the number of individuals who had at least one secondary healthcare visit within a 12-month period where that diagnosis was assigned as a main diagnosis as a proportion of the total number of individuals who were retained in the cohort.

A multinomial logistic regression model was conducted using the ‘nnet’ package to examine associations between sociodemographic, work disability and clinical factors at baseline and outcome status at the 9-year follow-up (healthcare utilisation for psychotic disorders vs healthcare utilisation for other psychiatric disorders vs no healthcare use for psychiatric disorders vs censored). Calendar year at cohort entry was included as a covariate to account for potential changes in diagnostic practices and clinical services. All predictor variables were entered into the model simultaneously, such that each was mutually adjusted for every other variable in the model, yielding adjusted ORs (aORs) and 95% CIs. Postestimation commands were performed using the ‘effects’ package to derive predicted probabilities, with 95% CIs, each of the four outcome groups at follow-up for all predictor variables. Predicted probabilities were then plotted for all predictors of interest (ie, all variables except year of cohort entry), for each outcome separately, ordered according to their ability to predict healthcare use for psychotic disorders at follow-up (highest to lowest).

## Results

### Sample characteristics

In total, 7733 individuals were treated for FEP in secondary healthcare from 2006 to 2013, of whom 7073 (91.5%) were followed for 9 years and 660 were lost to follow-up (407 died and 253 emigrated). Baseline characteristics are shown in table 1 by outcome group status. Across all outcome groups, the majority were male, born in Sweden, unmarried/without a partner and resided in cities. Only a small proportion (3.6%–5.8%) had experienced over 90 days of sickness absence in the years prior to their first diagnosis, while around 1 in 10 had been granted disability pension. Regarding treatments prior to FEP onset, 45.5%–62.8% had used secondary healthcare for other psychiatric disorders in the previous 3 years and 28.7%–47.2% had purchased any psychotropic medication in the 6 months prior to baseline. In terms of diagnosis at first contact (recorded at discharge for inpatient treatments and at the first physician visit for outpatient treatments), individuals diagnosed with other psychotic disorders (ICD-10 codes: F21–F22, F24–29) formed the majority across all outcome groups. Between 45.2% and 60.2% of individuals received their first psychotic disorder diagnosis in inpatient settings.

**Table 1:** Baseline characteristics of 7733 individuals who received a first diagnosis of psychotic disorder in secondary care between 2006 and 2013, according to follow-up status at 9 years

Baseline characteristics	Psychotic disorder(n=2455)		Other psychiatric disorder(n=1860)		No psychiatric disorder(n=2758)		Censored(n=660)	
Age category (years)*								
18–23	878	(35.8)	792	(42.6)	999	(36.2)	204	(30.9)
24–29	892	(36.3)	602	(32.4)	891	(32.3)	239	(36.2)
30–35	685	(27.9)	466	(25.1)	868	(31.5)	217	(32.9)
Sex†								
Female	811	(33.0)	819	(44.0)	1003	(36.4)	181	(27.4)
Male	1644	(67.0)	1041	(56.0)	1755	(63.6)	479	(72.6)
Country of birth†								
Sweden	1817	(74.0)	1531	(82.3)	2152	(78.0)	464	(70.3)
Elsewhere	638	(26.0)	329	(17.7)	606	(22.0)	196	(29.7)
Family situation†								
Other	2271	(92.5)	1684	(90.5)	2440	(88.5)	591	(89.5)
Married/partnered	184	(7.5)	176	(9.5)	318	(11.5)	69	(10.5)
Type of residence area†								
City	1271	(51.8)	830	(44.6)	1263	(45.8)	314	(47.6)
Town/suburb	830	(33.8)	731	(39.3)	1058	(38.4)	260	(39.4)
Rural area	354	(14.4)	299	(16.1)	437	(15.8)	86	(13.0)
Level of education†								
Compulsory	1161	(47.3)	1026	(55.2)	1347	(48.8)	369	(55.9)
High school	802	(32.7)	526	(28.3)	828	(30.0)	166	(25.2)
University	492	(20.0)	308	(16.6)	583	(21.1)	125	(18.9)
Days of unemployment†								
None	1641	(66.8)	1366	(73.4)	2027	(73.5)	470	(71.2)
Any	814	(33.2)	494	(26.6)	731	(26.5)	190	(28.8)
Days of sickness absence†								
None	2284	(93.0)	1686	(90.6)	2551	(92.5)	599	(90.8)
1–90	83	(3.4)	67	(3.6)	97	(3.5)	25	(3.8)
>90	88	(3.6)	107	(5.8)	110	(4.0)	36	(5.5)
Days of disability pension†								
None	2173	(88.5)	1557	(83.7)	2476	(89.8)	582	(88.2)
Any	282	(11.5)	303	(16.3)	282	(10.2)	78	(11.8)
Prior treatment for psychiatric disorder‡								
No	1323	(53.9)	691	(37.2)	1502	(54.5)	290	(43.9)
Yes	1132	(46.1)	1169	(62.8)	1256	(45.5)	370	(56.1)
Prior psychotropic medication§								
No	1751	(71.3)	982	(52.8)	1811	(65.7)	404	(61.2)
Yes	704	(28.7)	878	(47.2)	947	(34.3)	256	(38.8)
Diagnosis at first contact								
Other psychotic disorder	1445	(58.9)	1075	(57.8)	1542	(55.9)	374	(56.7)
Schizophrenia	317	(12.9)	109	(5.9)	166	(6.0)	42	(6.4)
Acute/transient psychotic disorder	693	(28.2)	676	(36.3)	1050	(38.1)	244	(37.0)
Treatment setting at first contact								
Outpatient	976	(39.8)	919	(49.4)	1511	(54.8)	275	(41.7)
Inpatient	1479	(60.2)	941	(50.6)	1247	(45.2)	385	(58.3)
Cohort entry year								
2006	337	(13.7)	237	(12.7)	318	(11.5)	70	(10.6)
2007	315	(12.8)	203	(10.9)	285	(10.3)	77	(11.7)
2008	267	(10.9)	247	(13.3)	356	(12.9)	77	(11.7)
2009	287	(11.7)	233	(12.5)	349	(12.7)	88	(13.3)
2010	291	(11.9)	235	(12.6)	340	(12.3)	84	(12.7)
2011	291	(11.9)	251	(13.5)	352	(12.8)	75	(11.4)
2012	324	(13.2)	222	(11.9)	392	(14.2)	77	(11.7)
2013	343	(14.0)	232	(12.5)	366	(13.3)	112	(17.0)

Values are numbers (percentages).

* Measured during year of cohort entry.

† Measured on 31 December in the calendar year prior to cohort entry.

‡ Measured in the three3 relative years (1080 days) prior to cohort entry date.

§ Measured in the 6 months (180 days) prior to cohort entry date.

### Healthcare use by diagnostic group at each year of follow-up

**Figure 1:**
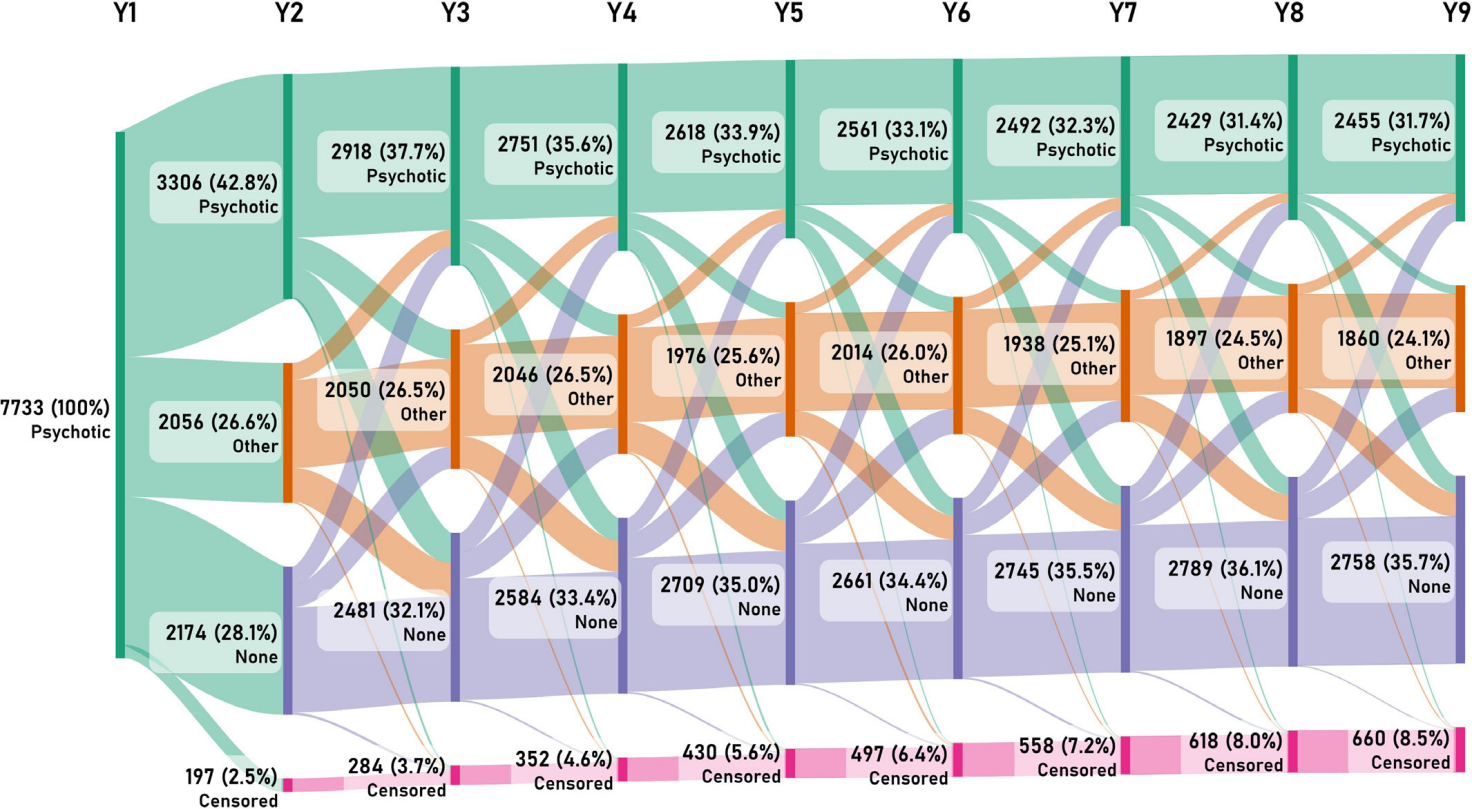
Sankey diagram showing the patterns of healthcare utilisation for psychiatric diagnostic categories (psychotic disorder vs other psychiatric disorder vs no psychiatric disorder vs censored) at each year after the first episode of psychosis.

Patterns of secondary healthcare utilisation at each year of follow-up are shown by diagnostic group in figure 1 for the entire cohort over the 9-year follow-up period. The 12-month period prevalence of healthcare use for psychotic disorders was 42.8% at year 2, which decreased to 37.7% at year 3, and then reduced by a further 1%–2% per year until reaching 31.7% at year 9. In contrast, 26.6% of individuals with an initial FEP diagnosis received a non-psychotic psychiatric disorder as their main diagnosis after the first year of follow-up, a proportion which remained relatively stable throughout the 9-year period. At the year 2 and year 3 follow-ups, 28.1% and 32.1% of individuals, respectively, had no secondary care contacts where a main diagnosis of psychiatric disorder was received, with this group accounting for 35.7% of the total sample by the final year. The number of individuals censored due to death/emigration increased by 0.5%–1.0% at each year of follow-up.

In supplementary analyses, we examined healthcare use for individual psychiatric diagnostic categories (online supplemental figure 1), where individuals could receive more than one diagnosis, with no hierarchy applied. The most common psychiatric diagnoses received in secondary healthcare at baseline were acute/transient psychotic disorders and other psychotic disorders. However, these numbers fell sharply within the first 3–4 years, and in the case of acute/transient psychotic disorders, remained relatively stable thereafter, whereas diagnoses of other psychotic disorders increased again after year 4, showing a second peak at around year 6. In contrast, the number of individuals diagnosed with schizophrenia was remarkably stable over the entire 9-year follow-up period. With regards to other psychiatric disorders, substance use, depressive and anxiety disorders were the most common and showed a gradual decline over the study period, whereas the number of individuals treated for bipolar disorder remained stable. Contacts for stress-related disorders were the least prevalent of the examined diagnostic groups throughout the study period.

### Associations of baseline factors with healthcare use at the 9-year follow-up

Results of the multinomial logistic regression model examining associations between baseline factors and outcome status at 9 years are shown in table 2 for all six pairwise comparisons (psychotic disorders vs other psychiatric disorders, psychotic disorders vs no disorder, other psychiatric disorders vs no disorder, psychotic disorders vs censored, other psychiatric disorders vs censored, no disorder vs censored). All baseline factors of interest were statistically significant in at least one pairwise comparison when mutually adjusted for all other variables in the model except for sickness absence. Several baseline factors, including being born outside of Sweden, completing high school education, unemployment in the previous year, having an initial diagnosis of schizophrenia and first diagnosis of FEP in inpatient settings, were significantly more common among those who used secondary healthcare for psychotic disorders at follow-up when compared with both those treated for other psychiatric disorders and those with no main diagnosis of a psychiatric disorder (aOR range: 1.17–2.18). In contrast, being married/partnered, living in towns/suburbs, purchases of psychotropic medication in the 6 months prior to baseline and receiving an initial diagnosis of acute/transient psychotic disorder were associated with significantly lower odds of using healthcare services for psychotic disorders at follow-up (aOR range: 0.60–0.85) when compared with the other psychiatric disorder and no psychiatric disorder groups. Compared with those lost to follow-up, individuals in the three other outcome groups were significantly less likely to be male and born outside of Sweden (aOR range: 0.48–0.80).

**Table 2:** Adjusted multinomial logistic regression models examining associations of demographic, work disability and clinical factors with outcome group at the 9-year follow-up among 7733 individuals who received a first diagnosis of psychotic disorder from 2006 to 2013

	Psychotic vs other psychiatric disorder (ref)	Psychotic vs no psychiatric disorder (ref)	Other vs no psychiatric disorder (ref)	Psychotic vs censored (ref)	Other psychiatric disorder vs censored (ref)	No psychiatric disorder vs censored (ref)
	aOR(95% CI)	aOR(95% CI)	aOR(95% CI)	aOR(95% CI)	aOR(95% CI)	aOR(95% CI)
Age category (years)*						
18–23	1.00 (reference)	1.00 (reference)	1.00 (reference)	1.00 (reference)	1.00 (reference)	1.00 (reference)
24–29	**1.32(1.13 to1.54**)	1.08 (0.94 to 1.24)	**0.82(0.70 to0.95**)	0.81 (0.65 to 1.01)	**0.62(0.49 to0.78**)	**0.75(0.60 to0.94**)
30–35	**1.39(1.16 to1.66**)	0.88 (0.76 to 1.03)	**0.64(0.54 to0.76**)	**0.71(0.56 to0.91**)	**0.51(0.40 to0.66**)	0.80 (0.63 to 1.02)
Gender†						
Female	1.00 (reference)	1.00 (reference)	1.00 (reference)	1.00 (reference)	1.00 (reference)	1.00 (reference)
Male	**1.43(1.26 to1.64**)	1.06 (0.94 to 1.20)	**0.74(0.65 to0.84**)	**0.69(0.57 to0.84**)	**0.48(0.39 to0.59**)	**0.65(0.53 to0.79**)
Country of birth†						
Sweden	1.00 (reference)	1.00 (reference)	1.00 (reference)	1.00 (reference)	1.00 (reference)	1.00 (reference)
Elsewhere	**1.39(1.19 to1.64**)	**1.19(1.04 to1.36**)	**0.85(0.73 to1.00**)	**0.80(0.65 to0.98**)	**0.57(0.46 to0.71**)	**0.67(0.55 to0.82**)
Family situation†						
Other	1.00 (reference)	1.00 (reference)	1.00 (reference)	1.00 (reference)	1.00 (reference)	1.00 (reference)
Married/partnered	**0.76(0.60 to0.96**)	**0.74(0.60 to0.91**)	0.97 (0.79 to 1.21)	0.83 (0.61 to 1.13)	1.09 (0.79 to 1.51)	1.12 (0.83 to 1.52)
Type of residence area†						
City	1.00 (reference)	1.00 (reference)	1.00 (reference)	1.00 (reference)	1.00 (reference)	1.00 (reference)
Town/suburb	**0.84(0.73 to0.96**)	**0.81(0.72 to0.92**)	0.97 (0.85 to 1.11)	**0.79(0.66 to0.96**)	0.95 (0.78 to 1.16)	0.98 (0.81 to 1.18)
Rural area	0.88 (0.73 to 1.06)	0.86 (0.73 to 1.02)	0.98 (0.82 to 1.17)	1.01 (0.77 to 1.32)	1.14 (0.86 to 1.51)	1.17 (0.89 to 1.53)
Level of education†						
Compulsory	1.00 (reference)	1.00 (reference)	1.00 (reference)	1.00 (reference)	1.00 (reference)	1.00 (reference)
High school	**1.32(1.14 to1.52**)	**1.17(1.02 to1.33**)	0.89 (0.77 to 1.03)	**1.54(1.24 to1.90**)	1.17 (0.94 to 1.45)	**1.31(1.07 to1.62**)
University	**1.22(1.01 to1.47**)	1.05 (0.89 to 1.23)	0.86 (0.72 to 1.03)	1.28 (0.99 to 1.64)	1.04 (0.80 to 1.36)	1.22 (0.95 to 1.56)
Days of unemployment†						
None	1.00 (reference)	1.00 (reference)	1.00 (reference)	1.00 (reference)	1.00 (reference)	1.00 (reference)
Any	**1.17(1.01 to1.35**)	**1.34(1.19 to1.52**)	1.15 (1.00 to 1.32)	**1.29(1.06 to1.57**)	1.11 (0.90 to 1.36)	0.96 (0.79 to 1.17)
Days of sickness absence†						
None	1.00 (reference)	1.00 (reference)	1.00 (reference)	1.00 (reference)	1.00 (reference)	1.00 (reference)
1–90	1.09 (0.78 to 1.54)	1.15 (0.84 to 1.57)	1.05 (0.76 to 1.46)	0.97 (0.61 to 1.56)	0.89 (0.55 to 1.44)	0.85 (0.53 to 1.34)
>90	0.74 (0.55 to 1.01)	0.99 (0.73 to 1.33)	1.33 (1.00 to 1.77)	0.77 (0.51 to 1.17)	1.04 (0.69 to 1.56)	0.78 (0.52 to 1.17)
Days of disability pension†						
None	1.00 (reference)	1.00 (reference)	1.00 (reference)	1.00 (reference)	1.00 (reference)	1.00 (reference)
Any	**0.72(0.59 to0.88**)	1.17 (0.97 to 1.42)	**1.62(1.34 to1.97**)	1.10 (0.82 to 1.46)	**1.52(1.13 to2.03**)	0.93 (0.70 to 1.25)
Prior treatment for psychiatric disorder‡						
No	1.00 (reference)	1.00 (reference)	1.00 (reference)	1.00 (reference)	1.00 (reference)	1.00 (reference)
Yes	**0.66(0.58 to0.76**)	1.12 (0.99 to 1.26)	**1.69(1.48 to1.93**)	**0.76(0.63 to0.91**)	1.15 (0.94 to 1.40)	**0.68(0.56 to0.82**)
Prior psychotropic medication§						
No	1.00 (reference)	1.00 (reference)	1.00 (reference)	1.00 (reference)	1.00 (reference)	1.00 (reference)
Yes	**0.60(0.52 to0.69**)	**0.85(0.74 to0.96**)	**1.40(1.23 to1.60**)	**0.71(0.58 to0.86**)	1.17 (0.96 to 1.43)	0.84 (0.69 to 1.01)
Diagnosis at first contact						
Other psychotic disorder	1.00 (reference)	1.00 (reference)	1.00 (reference)	1.00 (reference)	1.00 (reference)	1.00 (reference)
Schizophrenia	**2.18(1.71 to2.78**)	**1.95(1.58 to2.40**)	0.89 (0.69 to 1.16)	**1.96(1.39 to2.78**)	0.90 (0.61 to 1.32)	1.01 (0.70 to 1.45)
Acute/transient	**0.72(0.62 to0.82**)	**0.66(0.58 to0.74**)	0.92 (0.80 to 1.04)	**0.71(0.59 to0.86**)	0.99 (0.82 to 1.21)	1.08 (0.90 to 1.31)
Setting at first contact						
Outpatient	1.00 (reference)	1.00 (reference)	1.00 (reference)	1.00 (reference)	1.00 (reference)	1.00 (reference)
Inpatient	**1.39(1.22 to1.58**)	**1.96(1.75 to2.20**)	**1.41(1.25 to1.60**)	1.07 (0.90 to 1.28)	**0.77(0.64 to0.93**)	**0.55(0.46 to0.65**)
Cohort entry year						
2006	1.00 (reference)	1.00 (reference)	1.00 (reference)	1.00 (reference)	1.00 (reference)	1.00 (reference)
2007	1.11 (0.87 to 1.43)	1.05 (0.84 to 1.32)	0.94 (0.73 to 1.21)	0.86 (0.60 to 1.24)	0.78 (0.53 to 1.13)	0.82 (0.57 to 1.18)
2008	0.81 (0.63 to 1.04)	**0.73(0.58 to0.91**)	0.90 (0.71 to 1.14)	0.73 (0.51 to 1.06)	0.90 (0.62 to 1.31)	1.00 (0.70 to 1.44)
2009	0.92 (0.72 to 1.18)	0.80 (0.64 to 1.01)	0.87 (0.68 to 1.11)	0.71 (0.50 to 1.01)	0.77 (0.53 to 1.11)	0.89 (0.62 to 1.26)
2010	0.94 (0.73 to 1.20)	0.84 (0.67 to 1.05)	0.90 (0.70 to 1.14)	0.76 (0.53 to 1.09)	0.81 (0.56 to 1.18)	0.91 (0.63 to 1.29)
2011	0.88 (0.69 to 1.13)	**0.79(0.63 to0.99**)	0.90 (0.71 to 1.15)	0.84 (0.58 to 1.21)	0.96 (0.66 to 1.39)	1.06 (0.74 to 1.52)
2012	1.07 (0.83 to 1.37)	**0.79(0.63 to0.98**)	**0.74(0.58 to0.94**)	0.92 (0.64 to 1.33)	0.86 (0.59 to 1.26)	1.18 (0.82 to 1.69)
2013	1.12 (0.88 to 1.43)	0.93 (0.74 to 1.15)	0.83 (0.65 to 1.05)	**0.66(0.47 to0.93**)	**0.59(0.41 to0.85**)	0.72 (0.51 to 1.01)

* Measured during year of cohort entry.

† Measured on 31 December in the calendar year prior to cohort entry.

‡ Measured in the three3 relative years (1080 days) prior to cohort entry date.

§ Measured in the 6 months (180 days) prior to cohort entry date.

aOR, adjusted ORbold fontstatistically significant at the p<0.05 levelref, reference group

### Predicted probabilities of healthcare use at the 9-year follow-up

**Figure 2:**
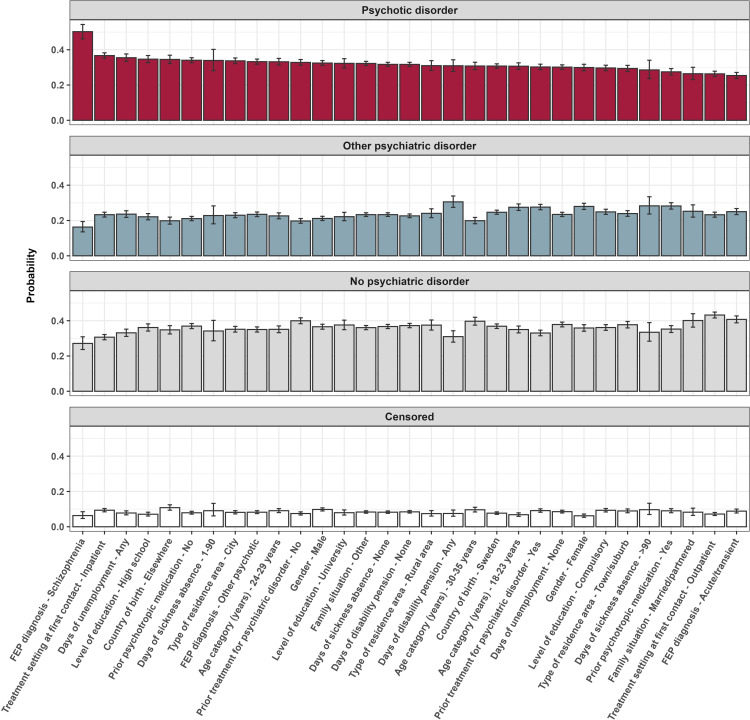
Predicted probabilities, with 95% CIs, for healthcare use for psychotic disorder, other psychiatric disorder, no psychiatric disorder and censored at the 9-year follow-up. FEP, first episode of psychosis.

Figure 2 shows the predicted probabilities of each outcome at the 9-year follow-up, for all baseline factors of interest, as derived from the multinomial logistic regression model. Having an initial diagnosis of schizophrenia was associated with highest probability of secondary healthcare use for psychotic disorders at the 9-year follow-up (0.50, 95% CI (0.46 to 0.54)), followed by first contact for FEP in inpatient settings (0.37, 95% CI (0.35 to 0.38)), prior unemployment (0.35, 95% CI (0.33 to 0.38)), completing high school education (0.35, 95% CI (0.33 to 0.37)) and being born outside of Sweden (0.35, 95% CI (0.32 to 0.37)). In contrast, the factors associated with highest probabilities for healthcare use for other psychiatric disorders were receipt of disability pension (0.31, 95% CI (0.27 to 0.34)), >90 days of sickness absence (0.28, 95% CI (0.24 to 0.33)), prior purchases of psychotropic medication (0.28, 95% CI (0.26 to 0.30)), female gender (0.28, 95% CI (0.26 to 0.30)) and previous treatment for other psychiatric disorders (0.28, 95% CI (0.26 to 0.29)). First treatment for FEP in outpatient settings (0.43, 95% CI (0.42 to 0.45)), having an initial diagnosis of acute/transient psychotic disorder (0.41, 95% CI (0.39 to 0.43)), being married/partnered (0.40, 95% CI (0.36 to 0.44)), having no history of treatment for psychiatric disorders (0.40, 95% CI (0.38 to 0.42)) and age 30–35 years at FEP onset (0.40, 95% CI (0.37 to 0.42)) were associated with the highest probabilities of no secondary healthcare use for psychiatric disorders at 9 years postdiagnosis. Baseline factors most strongly associated with being censored due to death/emigration by the end of the 9-year follow-up included being born outside of Sweden (0.11, 95% CI (0.09 to 0.12)), male gender (0.10, 95% CI (0.09 to 0.11)), having >90 days of sickness absence (0.10, 95% CI (0.07 to 0.13)), age 30–35 years (0.10, 95% CI (0.08 to 0.11)) and receiving the first psychotic disorder diagnosis in inpatient settings (0.09, 95% CI (0.08 to 0.10)).

## Discussion

In this population-based cohort study of all individuals aged 18–35 years diagnosed with FEP between 2006 and 2013 in Sweden, we observed that 32% were treated in secondary care for psychotic disorders at the 9-year follow-up, while a substantial proportion (24%) were treated for other psychiatric disorders. Having an initial diagnosis of schizophrenia, first contact for FEP in inpatient settings, prior unemployment, completing high school education and being born outside of Sweden were associated with highest probabilities of healthcare use for psychotic disorders at 9 years after the initial diagnosis.

Overall, around 56% of individuals in this FEP cohort utilised secondary healthcare for psychiatric disorders (psychotic or other disorders) 9 years after their first diagnosis. Our measure of healthcare use included both inpatient episodes and outpatient contacts, occurring within the 12 months prior to the 9-year follow-up. As such, our results cannot be compared with previous studies examining hospital admissions occurring at any point after the first psychotic episode.^11^,^13^ If we consider healthcare contacts for psychotic disorders specifically, the proportion of individuals who had discontinuous use of healthcare services for psychotic disorders over the entire 9-year period (68%) is much higher than the 21% recovery rate reported among individuals with non-affective FEP.^6^ However, it is important to note that as our outcome did not include primary care contacts or purchases of antipsychotic medication, we will have underestimated the proportion of individuals who were treated for psychotic disorder. Moreover, use of healthcare services is a marker of adequate and accessible service provision as well as ongoing illness. As such, there may be individuals who were actively unwell who, for various reasons, were unable to access treatment.

We next investigated factors associated with healthcare use for psychotic disorders and other psychiatric disorders at the 9-year follow-up. Here, we focused on factors measured at baseline (FEP diagnosis) given that (a) most covariates were time-invariant (eg, sex, country of birth) or measured at baseline because they provide important information regarding the mode of onset (eg, age at diagnosis, initial diagnosis, healthcare setting at first contact) and (b) factors that could vary over time are likely to be serially correlated, which can pose problems for model parameter estimation.^24^ In terms of prognostic factors, having an initial diagnosis of schizophrenia was associated with the highest probability of secondary healthcare use for psychotic disorders at the 9-year follow-up: after adjusting for all other factors, 50% of individuals with a baseline diagnosis of schizophrenia were predicted to have this outcome compared with 33% of those with other psychotic disorders and 25% of individuals with acute and transient psychotic disorders. This finding is consistent with the early Kraepelinian descriptions, in which schizophrenia (originally termed as ‘dementia praecox’) was delineated from other psychotic disorders on the basis that it was more commonly characterised by a chronic course and cognitive deterioration.^25^ Indeed, drawing on these early observations, it would be interesting in future studies to investigate whether patterns of long-term healthcare use differ among those with first episode non-affective versus affective psychotic disorders. Receiving the first diagnosis of psychotic disorder in inpatient settings was also associated with a high probability of secondary healthcare use for psychotic disorders at the 9-year follow-up (0.37). Potentially, individuals who first present with FEP to inpatient settings may have experienced a longer duration of untreated psychosis, a factor that has been consistently associated with poorer clinical and functional outcomes.^26^ Sociodemographic factors including, prior unemployment, high school education and being born outside of Sweden were also associated with higher probabilities of healthcare use for psychotic disorders at follow-up. The effect of country of birth concurs with our previous studies from this cohort, where we observed that migrant groups spent more days in hospital for psychotic disorders during the first 5 years of illness than their native-born peers.^15^ However, the findings for education were unexpected. High school and, to a lesser extent, university level education were both associated with a higher likelihood of being treated for psychotic disorders in secondary care at follow-up. The fact that we did not see the same pattern when comparing healthcare use for other psychiatric disorders versus no healthcare use for psychiatric disorders, suggests that this finding cannot be explained by individuals with higher education levels having better access to healthcare. Replication of these findings is needed to determine whether there is a paradoxical effect of higher education on long-term outcomes for people with psychosis.

A substantial proportion of people using secondary healthcare at the end of the 9-year follow-up received a primary diagnosis of a non-psychotic psychiatric disorder (43%). When we examined individual diagnostic categories, we found that substance use disorders were the most common, followed by anxiety and depressive disorders. One interpretation of this finding is that psychotic disorders, when examined collectively, have only moderate diagnostic stability. Indeed, stability estimates have been found to vary substantially across individual psychotic disorders, ranging from as high as 0.90 for schizophrenia to as low as 0.36 for psychosis not otherwise specified.^27^ An alternative interpretation is that this group includes individuals who made a full or partial recovery from their psychotic illness but continued to access treatment for other, potentially pre-existing, psychiatric disorders. Consistent with this interpretation, this group were more likely to have purchased psychotropic medication in the 6 months prior to cohort entry and to have been treated in secondary healthcare for other psychiatric disorders in the previous 3 years. Given that schizophrenia is the most costly^28^ and stigmatised^29^ of all psychiatric disorders, receiving a non-psychotic disorder diagnosis could be considered a good outcome from both an economic and patient perspective.

To our knowledge, this is the first study to investigate the impact of prior work disability on long-term healthcare use in an FEP population. As we might expect, measures of work disability were found to be important predictors of healthcare use for other psychiatric disorders. Indeed, among all factors examined, receipt of disability pension in the year prior to FEP onset was associated with the highest probability of secondary healthcare use for other psychiatric disorders at follow-up with long-term (>90 days) sickness absence ranked second. Interestingly, receipt of disability pension was associated with a lower likelihood of being treated for psychotic disorder at follow-up when this outcome was compared with other psychiatric disorders. Given that individuals who utilised secondary healthcare for other psychiatric disorders at follow-up were more likely to have been treated for these disorders in the 3 years prior to FEP onset, days of disability pension in the year prior to FEP may be attributable to these prior psychiatric disorders.

### Limitations

Our use of national register data means that our findings are relatively robust to selection and attrition bias, and we accounted for the latter by including those lost to follow-up (8.5% by the end of year 9) as a separate outcome group; however, due to small numbers, we were unable to examine the characteristics of those who died versus emigrated. Moreover, by linking multiple registers, we were able to identify several sociodemographic, work disability and clinical factors at baseline that were independently associated with healthcare use at the 9-year follow-up. However, some limitations must be noted. First, the FEP population was defined as individuals who had been resident in Sweden for 3 full years and had no prior treatments for any non-affective psychotic disorder in secondary care (where this was the main diagnosis) in this period. As such, our cohort might include those treated for psychosis in secondary care before this time period. However, given that 22% of the original cohort were born outside of Sweden (and were therefore missing data on healthcare use prior to migration), this restriction was necessary to reduce the risk of differential misclassification of first-episode status. Second, as our study focused on secondary healthcare use, we did not examine primary care contacts (either prior to, or after, diagnosis); as such, healthcare use for non-psychotic psychiatric disorders in particular will be underestimated as these disorders are commonly treated exclusively in primary care. In contrast, it is very unusual for individuals with a primary diagnosis of a psychotic disorder to be treated in primary care only. Third, the individuals who were categorised as having not used secondary healthcare for psychiatric disorders at follow-up are likely to be a heterogeneous group, in that they include those who made a full recovery from psychosis as well as individuals with psychiatric disorders who were treated exclusively in primary care, and those who continued to experience active illness but were unable to access healthcare. As such, we cannot consider this to be a good outcome within this cohort. Fourth, our use of registers limits our ability to examine other potentially important clinical predictors of long-term healthcare use such as measures of symptoms, cognition and social functioning at baseline. Finally, as our cohort included individuals in Sweden aged 18–35 years, who developed psychosis between 2006 and 2013, our findings may have limited generalisability to other countries, individuals who develop psychosis in later life and current clinical practices. With regards to the latter point, although psychosis early intervention services have been implemented in many countries over the past two decades, these specialist services remain scarce in Sweden,^30^ as such, it is unlikely that clinical practice for this population has changed radically during the study period. Importantly, year of cohort entry was included as a covariate and there were no consistent patterns that emerged across time (as we might have seen if there were fundamental changes in healthcare provision).

### Clinical and research implications

Our finding that just over half of individuals presenting with FEP continued to receive treatment for psychiatric disorders 9 years later highlights the long-term impact of these disorders at the personal, societal and economic level. Moreover, given that this population is known to be at elevated risk for a range of physical health conditions,^31^ it is likely that the overall burden on healthcare services is even higher. The factors which we have shown to be associated with healthcare use for psychotic disorders specifically are important for patients and their families, clinicians and those responsible for planning healthcare provision. For example, clinical teams should consider that individuals with an initial diagnosis of schizophrenia, who received their first diagnosis in inpatient settings, and those born outside of the host country are more likely to require intensive treatment and additional support to facilitate remission and recovery from the initial psychotic episode. However, further research is needed to establish whether the associations that we have observed at the group level can be applied to the individual level. Here, analyses that incorporate longitudinal predictor variables (eg, dynamic prediction models) may achieve greater predictive accuracy than those based on baseline factors alone.

## supplementary material

10.1136/bmjment-2024-301248 online supplemental file 1

## Data Availability

Data may be obtained from a third party and are not publicly available.
