# Supplementary material for: Prevalence and predictors of healthcare use for psychiatric disorders at 9 years after a first episode of psychosis: a Swedish national cohort study
Source: BMJ Ment Health. 2025 Mar 26;28(1):e301248. doi: 10.1136/bmjment-2024-301248 (PMC11950954; doi:10.1136/bmjment-2024-301248)
Supplement: online supplemental file 1 [file bmjment-28-1-s001.docx]

**Supplementary Material**

van Deursen D, Mittendorfer-Rutz E, Taipale H, Pettersen E, McGuire P, Fusar-Poli P, Joyce DW, Albert N, Erlangsen A, Nordentoft M, Hjorthøj C, Cervenka S, Cullen AE. Prevalence and predictors of healthcare use for psychiatric disorders at nine years after a first episode of psychosis: A Swedish national cohort study. *BMJ Mental Health.*

**Supplemental Table 1** Sociodemographic, work disability, and clinical measures, definitions and data sources

| Variable | Definition | Register |
| --- | --- | --- |
| Sociodemographic |  |  |
| Age | Years of age during the calendar year of cohort entry, categorised as 18-23 years vs. 24-29 years vs. 30-35 years | LISA |
| Gender | Measured at birth, categorised as men vs. women | LISA |
| Country of birth | Categorised as Sweden vs. other | LISA |
|  |  |  |
| Family situation | Measured on the 31st of December in the year prior to cohort entry, categorised as singe vs. married/cohabiting | LISA |
| Type of residence region | Measured on the 31st of December in the year prior to cohort entry, categorised as cities vs. towns/suburbs vs. rural, according to EUROSTAT’s degree of urbanization [DEGURBA] classification of local administrative units | LISA |
| Level of education | Measured on the 31st of December in the year prior to cohort entry, categorised as compulsory vs. high school vs. college/university | LISA |
| Unemployment | Number of unemployment days during the calendar year prior to cohort entry, categorised as none vs. any | LISA |
| Work disability |  |  |
| Sickness absence | Gross sickness absence days in spells exceeding 14 days measured during the calendar year prior to cohort entry, categorised as 0 vs. 1-90 vs. >90 days | MiDAS |
| Disability pension | Receipt of disability pension during the calendar year prior to cohort entry, categorised as none vs. any | MiDAS |
| Clinical |  |  |
| Prior treatment for any non-psychotic psychiatric disorder | Any inpatient/specialist outpatient treatment with a main diagnosis of any non-psychotic psychiatric disorder (ICD-10 codes F00-F99, excluding F20-29) during the three relative years (1080 days) prior to cohort entry, categorised as no vs. yes | NPR |
| Prior psychotropic medication | Dispensations of any psychotropic medications, including antipsychotics, lithium, anxiolytics, benzodiazepines, hypnotics, Z drugs, antidepressants, and mood stabilisers (ATC codes N05A, N05B, N05C, N06A, N03AF01, N03AG01, N03AX09, and N05AN01) measured during the six months (180 days) prior to cohort entry, categorised as no vs. yes | PDR |
| Diagnosis at cohort entry | Diagnosis at first contact (assigned at discharge/contact for inpatient/outpatient treatments, respectively), categorised as schizophrenia (ICD-10: F20) vs. schizotypal disorder (ICD-10: F21) vs. delusional disorder (ICD-10: F22) vs. acute or transient psychotic disorder (ICD-10: F23) vs. other (ICD-10: F24-F29) | NPR |
| Treatment setting at first diagnosis | Treatment setting where first diagnosis of NAPD was received, categorised as outpatient vs. inpatient | NPR |
| Censoring variables |  |  |
| Death | Death during nine-year study period, categorised as no vs. yes | CDR |
| Emigration | Emigration during nine-year study period, categorised as no vs. yes | LISA |

Abbreviations: ATC: Anatomic Therapeutic Chemical; CDR: Cause of Death Register; ICD-10: International Classification of Diseases – version 10; LISA: Longitudinal Integration Database for Health Insurance and Labor Market Studies; MiDAS: Micro-Data for Analyses of Social Insurance; NAPD: non-affective psychotic disorder; NPR: National Patient Register; PDR: Prescribed Drug Register.


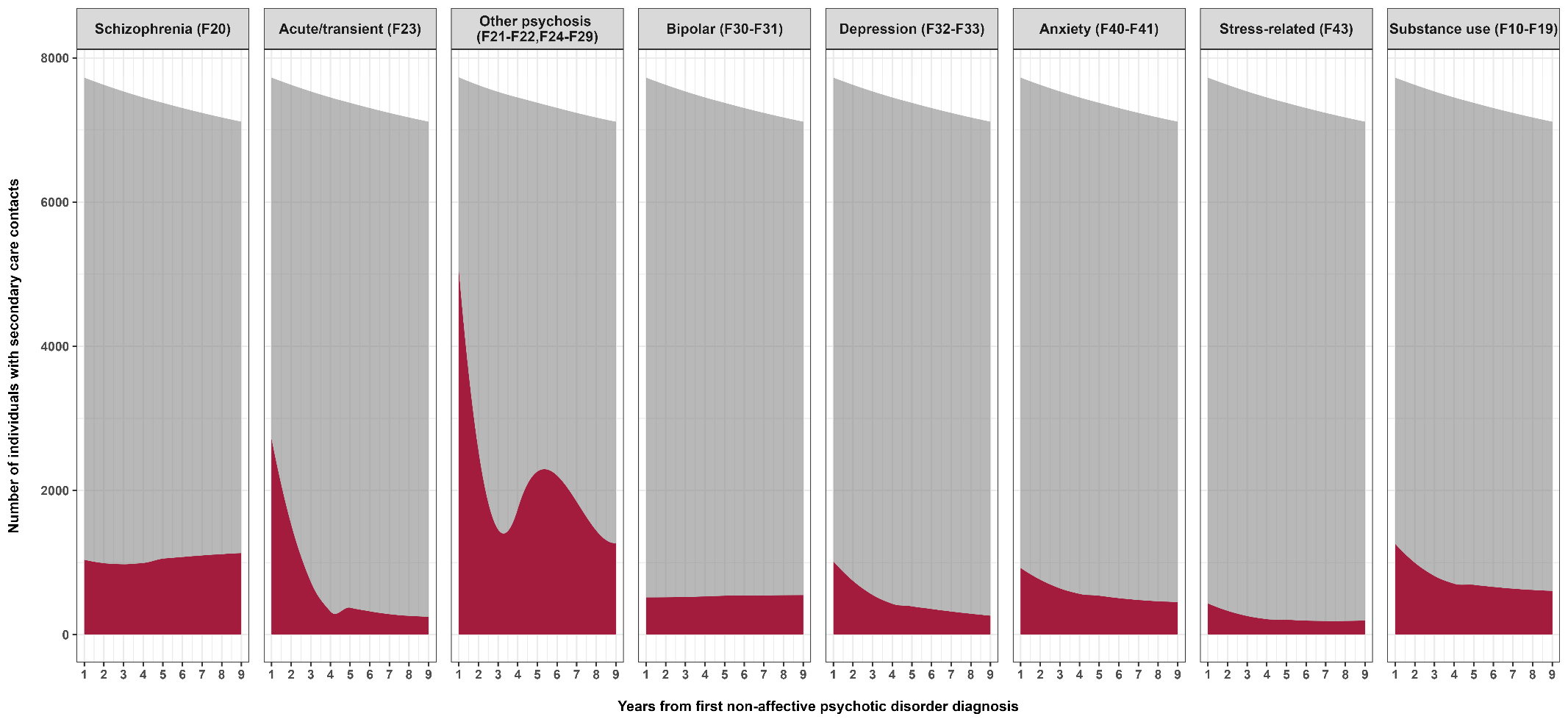


**Supplemental Figure 1** Partial density plots of individual diagnostic categories (ICD-10 codes) received in secondary healthcare at each year of follow-up after the first episode of psychosis. Plots show, for each diagnostic category, the number of individuals with at least one secondary healthcare contact where the corresponding diagnosis was given as the main diagnosis (red) relative to the total number of individuals who remained in the cohort at each year of follow-up (grey). Diagnostic categories are not mutually exclusive, that is, individuals could receive more than one diagnosis during each year of follow-up, with no hierarchy applied.
